# Characterization of oxidative stress in animal model of neonatal hypoxia

**DOI:** 10.1590/ACB361108

**Published:** 2021-12-17

**Authors:** Liliane de Mello Santos Bochenek, Eduardo Benedetti Parisotto, Evelyn de Andrade Salomão, Maria José Martins Maldonado, Iandara Schettert Silva

**Affiliations:** 1 Fellow Master degree. Postgraduate Program in Health and Development - Universidade Federal do Mato Grosso do Sul (UFMS) - Campo Grande (MS), Brazil.; 2 PhD. Associate Professor. Faculty of Pharmaceutical Sciences, Food and Nutrition (FACFAN) - Universidade Federal do Mato Grosso do Sul (UFMS) - Campo Grande (MS), Brazil.; 3 Fellow PhD degree. Postgraduate Program in Health and Development - Universidade Federal do Mato Grosso do Sul (UFMS) - Campo Grande (MS), Brazil.; 4 PhD. Associate Professor. Postgraduate Program in Health and Development - Universidade Federal do Mato Grosso do Sul (UFMS) - Campo Grande (MS), Brazil.

**Keywords:** Oxidative Stress, Hypoxia, Swine

## Abstract

**Purpose:**

To evaluate the oxidative stress in swine neonates submitted to hypoxia.

**Methods:**

Ten large white piglets, healthy newborns, of both sexes, were divided into two groups and submitted to an experimental hypoxia protocol with reduced inspired oxygen fraction. The hypoxia group, composed of six animals, was submitted to oxygen reduction for 180 min. The animals in the control group, n = 4, were handled and evaluated simultaneously, but without oxygen reduction.

**Results:**

180 min after the start of the hypoxic insult, a significant difference was observed in the oximetry, and heart rate of the hypoxia group was compared to the control group (p<0.05). There was no significant difference in the oxidative stress analyses. Reduced glutathione (GSH), superoxide dismutase (SOD), catalase (CAT), lipid peroxidation (TBARS), protein carbonyl (PC), and myeloperoxidase (MPO) in the piglets’ brain tissue were analyzed.

**Conclusions:**

Hypoxia causes adverse effects in swine neonates, although there is a natural physiological resistance of swine neonates to respond to this insult. Analyses of GSH, SOD, CAT, TBARS, MPO, and PC were tabulated and are presented as parameters for further studies to be carried out on an animal model of swine hypoxia.

## Introduction

Oxidative stress occurs due to an imbalance of oxidants in relation to antioxidants. At physiological levels, oxidants play a beneficial role in energy production, cell signaling and host defense. In excess, they can lead to pathological consequences for tissue and cell damage, disorders and diseases, resulting from the lack of antioxidant protection systems, or defects in the genes that regulate the antioxidant machines^1^,^2^.

Reactive oxygen species (ROS) have been implicated in the pathogenesis of hypoxic-ischemic injury leading to an increase in oxidative stress and causing significant damage to biological macromolecules such as proteins (degeneration of membrane proteins), lipids (lipid oxidation) and acids (DNA degeneration). No intervention after asphyxia in human birth has been shown to reduce encephalopathy and subsequent brain damage. However, from recent work in newborn and adult animals, there is compelling evidence that neuronal rescue therapy is effective even if applied after the insult^3^-^6^.

During the metabolism of oxygen and nitrogen, several toxic molecules are produced, such as ROS. Any species that uses oxygen during cell respiration or nitrogen, by breaking down dietary amino acids for energy, are subject to oxidative stress^3^,^7^,^8^.

Perinatal asphyxia resulting in hypoxic-ischemic encephalopathy (HIE) is the cause of several pathologies in neonates, especially preterm ones, and more than one million of them die. In Europe, complications of prematurity are the cause of more than half of deaths in the first year of life, and there are still clinical manifestations in adulthood in those who survive^2^.

Large animal models are an essential tool in the development of new rationale-based clinical therapies for preterm infants. The pig provides a relevant model for the study of premature human physiology and for the investigation of new therapies to improve outcomes^9^. The newborn piglet reaches a degree of maturity at birth similar to that of humans with regard to cardiovascular regulation. Therefore, this species has been considered an excellent subprimate’s laboratory model for comparison studies with human infants^10^.

Although the newborn piglet is an accepted large animal model of perinatal asphyxia/HIE, there is no consensus on the methodology of inducing perinatal asphyxia to produce clinically relevant HIE^11^.

Therefore, it is important to characterize what alterations are related to oxidative stress during hypoxia in the neonatal development of encephalopathy. This study intended to characterize oxidative stress in an experimental animal model compatible with the pathophysiological changes we see in neonates, in order to seek alternatives for the control of oxidative and neurological damage.

## Methods

This study was approved by the Ethics Committee on the Use of Animals (CEUA) of Universidade Federal de Mato Grosso do Sul (UFMS), protocol no. 1,088/2019.

Ten newborn pigs, large white, healthy, of both sexes, aged 48 hours old and having 1,470 g (± 0.24) of average weight. They were removed from the mother three hours before the start of the experiment. Animals were transported from a commercial farm in a warm and quiet environment to minimize stress. Piglets were fed with breast milk, every three hours, purchased from the farm. Upon arrival at the laboratory, they were evaluated and had their physiological data (heart rate, respiratory rate, mean arterial pressure, temperature, and oximetry), weight, and group identification recorded in the protocol.

The animals were randomly distributed into two groups: hypoxia group (HG=6) and control group (CG=4). Blood collection was performed before and after hypoxia induction/simulation, for arterial blood gas analysis, blood count and biochemical tests. A quantity of whole blood was collected in a tube containing ethylenediamine tetraacetic acid (EDTA) to carry out the blood test of the animals. Biochemical tests were performed on whole blood serum alanine transaminase (ALT, aminotransferase–AST, creatinine and urea).

The animals were euthanized after the experiment with the association of Thiopental (100 mg/kg) followed by potassium chloride (75 mg/kg), via intracardiac.

### Experimental protocol for hypoxia induction

The animals were placed, in pairs, in glass chambers (33 × 40 × 30 cm) with hermetic closing performed by a lid/box seal with silicone adhesive in combination with oxygen supplementation, according to the model described by Paiva. Control of the concentration of oxygen inside the chamber was performed with the aid of a calibrated flowmeter (capacity 15 L/min) fed by a medical grade oxygen cylinder.

After monitoring the resting state, the inspired oxygen fraction was reduced from 0.3 to 0.1 by reducing the tidal volume of oxygen, and the box was closed in order to limit gas exchange with the external environment, inducing normocapnic hypoxia for 180 min. Then, the initial gas volume was reestablished to record the physiological data of the animals (post insult).

The control group was manipulated and evaluated simultaneously with the other animals, but it had oxygen administered continuously, thus preventing the accumulation of carbon dioxide (CO_2_) and maintaining a constant desirable oxygen concentration (24%) to all animals in the set time of 180 min. After exposure to oxygen, the animals were removed from the box, reassessed for physiological parameters, and a new blood collection was performed.

Sixty min after the end of the insult, the animals were euthanized, and the brain was removed for analysis of oxidative stress markers. During the entire procedure, the animals remained awake. Two animals required sedation with 1% acepromazine (0.03 mL i.m.) for containment during blood sample collection, in order to reduce stress.

### Collection, preparation and analysis of brain tissue

The entire brain was collected after the animal’s death was confirmed. A longitudinal section of the brain was performed, ensuring that all structures were included (brain, hypothalamus, cerebellum, and brainstem).

The samples were macerated in two Falcon tubes (one with trichloroacetic acid – 12% TCA; and the other without reagent) and stored in liquid nitrogen (≅170°C). In the tube containing 12% TCA (tube 1), the brain samples were macerated and homogenized, thus obtaining a processed sample called acid extract, which was used to determine the content of reduced glutathione (GSH). The second brain sample (tube 2) was gradually thawed at temperature (ice and room temperature), macerated and homogenized in TRIS 300 mM HCl buffer, pH 7.4 (called lysis buffer). The content of this tissue homogenate was centrifuged at 3,000 g for 5 min, and the supernatant was used to perform the other oxidative stress markers (lipid peroxidation–TBARS, protein carbonyl–PC, myeloperoxidase–MPO, superoxide dismutase–SOD, and catalase–CAT). All oxidative stress markers were normalized through the quantification of total proteins in the samples, by the Bradford method, using an albumin standard curve. Assays were performed in duplicates.

### Oxidative stress assessment

Enzyme activities were measured as described:

- Reduced glutathione (non-protein thiols): The content of reduced GSH was evaluated in the acid extract obtained from the brain through the determination of non-protein thiols (GSH represents ≅ 95% of the total of these thiols). Twenty μL of 2.5 mM 2-nitrobenzoic acid (DTNB) was added in a 96-well plate containing 190 μL of 0.2 M potassium phosphate buffer pH 8 and 10 μL of the sample (brain acid extract). Subsequently, the plates were intermittently agitated, until the maximum formation of the yellow color thiolate anion (TNB) was obtained, measurable at 412 nm. Values measured in duplicate and expressed in μmol/mg protein;
- Superoxide dismutase (SOD): SOD activity was measured spectrophotometrically at 480 nm through the oxidation of adrenaline (change from pH 2 to pH 10), which forms the superoxide anion and a pink chromophore, the adrenochrome, in which the enzyme presents in the sample delays its formation. Values were expressed in terms of enzyme activity, that is, an arbitrary unit of SOD is defined as the amount of enzyme needed to halve the rate of adrenochrome formation. Therefore, the samples homogenized in lysis buffer were added to 96-well plates, in ascending order of volume (5, 10, 20, 40 μL), and the volume was made up to 200 μL with 50 mM glycine buffer, pH 10. In sequence, 5 μL of 60 mM adrenaline (approximately pH 2 in ice and amber bottle) was added. The speed of adrenochrome formation was monitored, and then the sample aliquot was added (5 to 40 μL), totaling 8 min. The curves made it possible to indirectly assess the enzymatic activity of SOD. Values were expressed in USOD/mg protein;
- Catalase (CAT): Catalase activity (CAT) was analyzed at 240 nm, quantifying the decrease in the level of H_2_O_2_ (expressed in mmol/min/g) in a 10 mM H_2_O_2_ solution. For the determination of superoxide dismutase (SOD) activity, the oxidation of epinephrine (pH 2 – pH 10.2), which produces a superoxide anion and a pink chromophore (expressed in USOD/g), was quantified at 480 nm.

The content of reduced GSH was evaluated in the acid extract obtained from the brain through the determination of non-protein thiols (GSH represents ≅95% of the total of these thiols), measurable at 412 nm.

Duplicate measured values were expressed in μmol/mg protein.

### Oxidative damage markers

- Lipid peroxidation (TBARS): The evaluation of lipid peroxidation in the brain was performed in duplicate, by detection at 535 nm of derivatives of their oxidation products, through substances that react with thiobarbituric acid (TBA)–TBARS, mainly MDA, producing a Shiff base of pink color. For this assay, in microtubes containing 500 μL of 12% TCA, 50 μL of the sample supernatant were added, homogenized in lysis buffer and centrifuged. Then, 450 μL of TRIS buffer (Tris HCl solution 7.4) were added and, finally, 500 μL of thiobarbituric acid (TBA) 0.73%. After vortex homogenization, incubation was carried out for 60 min at 100°C. Then, the material was cooled for 15 min at 4°C and centrifuged (5,000 g for 5 min). The supernatant was used for reading, and the results were expressed in mmol.mg;
- Protein carbonyls (PC): Oxidative damage to proteins was determined by protein carbonylation by spectrophotometric measurement. For this analysis, centrifuge microtubes containing 600 μL of 2,4-dinitrophenylhydrazine (DNPH) were used, and 100 μL of the homogenized and centrifuged sample were added. Subsequently, it was incubated for 1 h (at room temperature, protected from light, under agitation). Then, 600 μL of 20% TCA was added, followed by stirring and refrigeration (ice bath) for 15 min and centrifuged for 5 min at 800 g. The pellet formed was washed 3 times, followed by centrifugation for 5 min at 800 g, using 800 μL of ethanol:ethyl acetate (1:1). After the last wash, the excess of ethanol:ethyl acetate was removed with the aid of a cotton swab, and 800 μL of guanidine were added and incubated for 60 min at 37°C and then reading at 360 nm. The carbonyl protein concentration was expressed in nmol.mg of protein.

### Inflammatory marker

- Myeloperoxidase (MPO): Serum MPO activity was measured according to the method described by Rao et al.. First, samples were gradually thawed, and 20 μL were transferred to plates containing 180 μL of reaction medium (0.167 mg.mL^-1^ o-dianisidine 2HCl, 0.0005% H_2_O_2_, distilled H_2_O and 50 mM NaH_2_PO_4_). After 15 min of incubation at room temperature, the reaction was stopped with the addition of 30 μL of 1% sodium azide. After 10 min incubation at room temperature, optical density was measured at 450 nm in 96-well plates and compared to a standard curve of known MPO activities (0.7 to 140 mU.mL^-1^). Results were expressed in mU.mg.

### Statistical analysis

Student’s t test for independent samples was applied for the statistical evaluation of all mean and standard deviation (SD) differences belonging to the control (CG) and hypoxia groups (HG), admitting the minimum significance level of p<0.05. To verify the use of the t test, the Shapiro-Wilk test was previously performed, checking the normality of the samples. Descriptive statistics were used to describe the basic characteristics of the data in terms of mean value (mean) and SD. Analyses were performed using the BioEstat 5.3 statistical program.

## Results

The results of the physiological parameters, blood gases, and biochemical analysis of the animals according to the time of analysis and the belonging group (HG or CG) are shown in Table 1.

**Table 1:** Values listed as mean (± SD). Significant group differences: p values in bold. Results are presented as mean ± SD of the mean. Values in bold represent statistically significant differences (p<0.05). Comparison between hypoxia group and control group: Student’s t test.

Physiological measurements		Hypoxia group	Control group	p-value
		(n=6)	(n=4)	
Body weight (kg)		1.47 (± 0.22)	1.46 (± 0.30)	0.9566
Sex (male:female)		4:2	1:3	
HR (bpm)				
	Start	184.5 (± 57.74)	210 (± 39.64)	0.4552
	End	130.17 (± 39.34)	200.25 (± 34.57)	**0.0203**
RR (mpm)				
	Start	53.67 (± 34.89)	33 (± 7.57)	0.127
	End	44.33 (± 18.40)	33 (± 6.73)	0.1506
Temperature (°C)				
	Start	37.98 (± 1.55)	36.40 (± 1.23)	0.127
	End	36.63 (± 0.62)	37.23 (± 0.51)	0.1506
Mean arterial blood pressure (mmHg)				
	Start	103.67 (± 41.11)	127 (± 16.87)	0.2541
	End	88.17 (± 27.21)	110.5 (± 31.86)	0.2939
Oximetry (%)				
	Start	91.67 (± 5.82)	94.75 (± 2.75)	0.3582
	End	77.33 (± 6.53)	95.50 (± 3.7)	**0.0011**
pO_2_ (mmHg)				
	Start	37.82 (± 14.73)	33.85 (± 13.17)	0.6698
	End	32.33 (± 11.53)	18.53 (± 3.18)	**0.032**
pCO_2_ (mmHg)				
	Start	35.53 (± 4.69)	30.40 (± 1.80)	**0.0458**
	End	32.50 (± 8.88)	35.23 (± 3.33)	0.5164
HCO_3_ (mmol/L)				
	Start	22.22 (± 7.39)	20.20 (± 2.37)	0.5569
	End	19.37 (± 6.58)	22.35 (± 0.45)	0.3186
pH				
	Start	7.38 (± 0.14)	7.42 (± 0.04)	0.5373
	End	7.38 (± 0.08)	7.41 (± 0.05)	0.4476
Na (mmol/L)				
	Start	147.88 (± 6.25)	145 (± 2.62)	0.3478
	End	149.03 (± 4.92)	148.03 (± 2.58)	0.684
K (mmol/L)				
	Start	2.94 (± 0.68)	3.10 (± 0.59)	0.7147
	End	3.08 (± 0.48)	3.17 (± 0.36)	0.7672
iCA (mmol/L)				
	Start	0.85 (± 0.18)	0.89 (± 0.15)	0.696
	End	0.86 (± 0.21)	0.81 (± 0.07)	0.6538
Cl (mmol/L)				
	Start	107.97 (± 4.10)	107.50 (± 1.64)	0.8093
	End	112.97 (± 6.26)	107.98 (± 1.63)	0.2296
Glu (mg/dL)				
	Start	85.22 (± 26.33)	85.50 (± 12.71)	0.9825
	End	94.33(± 11)	76 (± 7.87)	**0.0154**
Lac (mmol/L)				
	Start	7.71 (± 3.98)	5.70 (± 1.07)	0.2852
	End	7.50 (± 5.11)	3.80 (± 0,18)	0.1366
ALT (U/L)				
	Start	32.24 (± 5.94)	24 (± 6.39)	0.0945
	End	30.48 (± 6.77)	23.73 (± 5.95)	0.1693
AST (U/L)				
	Start	49.92 (± 10.23)	40.15 (± 24.85)	0.5016
	End	50.02 (± 10.91)	29.15 (± 9.03)	**0.0188**
URE (mg/dL)				
	Start	25.88 (± 15.58)	19.50 (± 7.26)	0.4477
	End	29.17 (± 9.54)	22.20 (± 8.23)	0.2941
CRE (mg/dL)				
	Start	0.54 (± 0.13)	0.43 (± 0.15)	0.2763
	End	0.57 (± 0.05)	0.40 (± 0.07)	**0.0151**

SD: standard deviation; HR: heart rate; RR: respiratory rate; iCA: calcium; Glu: glucose; Lac: lactate; ALT:alanine transaminase; AST: aspartate aminotransferase; URE: urea; CRE: creatinine,

There was a significant difference in the oximetry and heart rate of the animal groups after the 180-min hypoxic insult. The measurement of AST and CRE also showed a significant difference after the experiment.

The results related to the analysis of oxidative stress, oxidative damage markers and inflammatory marker are presented in Table 2. There was no significant difference in the analyses (p>0.05).

**Table 2:** Values listed as mean (± SD). Results are presented as mean ± SDon of the mean.

	Hypoxia group	Control group	p-value
GSH	0.3481 ±0.0730	0.4113 ± 0.0890	0.2520
SOD	17.1663 ±9.7926	12.8420 ±2.7412	0.4223
CAT	6.9616 ±2.5582	5.0995 ±0.8949	0.2058
PC	0.00013 ±0.00002	0.00016 ±0.00004	0.0944
TBARS	0.0108 ±0.0017	0.0095 ±0.0021	0.3057
MPO	79.4125 ±47.2810	120.6752 ±56.4665	0.2404

SD: standard deviation; GSH: glutathione; SOD: superoxide dismutase; CAT: catalase; PC: protein carbonyl; TBARS: lipid peroxidation; MPO: myeloperoxidase.

## Discussion

The results found in our study present an overview of cerebral oxidative stress developed in piglets as an animal model of neonatal hypoxia. Although the sample size is the minimum necessary (respecting the principle of 3Rs), biochemical measurements and clinical characteristics of the animal’s impairment in face of the insult were observed. Similar to several related studies, rapid restoration of the piglet to hypoxic damage was observed. Schulz *et al*.^10^ report that, in experimental studies, work with “small samples” is common, especially in the case of newborns.

Changes in animals during the hypoxia period demonstrate clinical signs compatible with encephalopathy. In addition to the significant changes observed in heart rate and oximetry, changes in breathing, orientation, gait, reduced activity level and presence of pathological movements were also evidenced as described by other authors^13^.

Although a picture of metabolic acidosis in piglets is described to confirm the hypoxic lesion, several authors reiterate the tolerance to hypoxia in many animals^4^,^5^,^9^,^13^ and even report a brief recovery from the insult suffered^4^,^5^. In our study, no significant difference was observed in the pH of the animals (HG=7.38±0.08 and CG=7.41±0.05 after 180 min).

An animal model of disease, with piglet as the EHI model, is technically challenging to develop, and even replicate. The literature describes several ways to promote the insult, and all emphasize the animal’s resistance and short recovery to the condition, as well as the different factors that need to be taken into account when promoting hypoxia (hypoxia induction method, anesthesia, cord clamping umbilical, clamping of the carotid artery, and others).

Unlike adults and children after brain trauma, human newborns with neonatal encephalopathy are not routinely anesthetized. Depending on local practice, infants can be sedated during mechanical ventilation, and intensive care can receive anticonvulsant treatment if they develop clinical seizures^4^,^10^. After birth, the extrauterine environment conditions the newborn to a series of adaptations to life, which can generate or develop problems with sequelae that are difficult to reverse, such as those related to physical, cognitive, learning, and behavioral development^10^,^14^. Thus, our objective was to replicate the clinical routine and not anesthetize the animals, but promote mild sedation in order to provide well-being to the piglets during the experiment.

At physiological levels, oxidants play a beneficial role in the body in several vital functions, such as energy production and host defense. Excessive presence of oxidants (ROS) in relation to antioxidants is defined as oxidative stress that results in pathological changes in the body^1^,^7^. Thus, it is interesting to determine the antioxidant status, in individuals under different pathological situations, such as our model of neonatal hypoxia, through different parameters such as: lipid peroxidation, SOD, GSH, among others^8^.

The excessive production of ROS has been implicated as the main mediators of perinatal brain damage, especially in preterm infants, as there is potentially an imbalance between the generation of oxidant substances and the interaction of the antioxidant defense system, and it is important to know the oxidative status to control the progression of the disorder. Biological membranes are sensitive to lipid peroxidation induced by ROS, and the increase in lipid peroxidation will lead to several deleterious effects. Oxidative stress followed by apoptosis has been considered a critical step in neonatal brain injury^5^,^7^,^14^.

Compounds that can be modified by the action of free radicals, such as lipids and proteins, are also used as indirect measures of oxidative stress, as well as the enzymatic activities involved in cellular redox balance, such as SOD and CAT. Although GSH is not an enzyme, it acts in the conversion of superoxide radicals into peroxides^1^,^15^. Other biomarkers, called oxidative damage products, can also be measured and the main one is MDA, formed at the end of lipoperoxidation processes^7^.

The brain is one of the main organs affected by hypoxia in the neonatal period and one of the most vulnerable to oxidative stress. Therefore, it leads to compromised neurogenesis, mitochondrial damage, and neuroinflammation. The brain also accumulates high levels of transition metals, such as iron and copper, being an important organ to demonstrate changes in the antioxidant balance^2^.

To assess the effects of hypoxia on cerebral oxidative damage, we measured PC levels, a marker of ROS-induced protein damage, and TBARS (thiobarbituric acid reactive substances, especially malondialdehyde) a marker of lipid peroxidation. MPO is an enzyme present in neutrophils which is associated with the phagocytic process, acting as an important inflammatory marker in several pathologies^15^.

MPO has been reported as an important parameter in the early assessment of several pathological conditions. Inflammatory activation precedes the onset of a diagnosis-confirmed coronary disease, which could indicate MPO as an earlier marker for possible inflammatory vascular changes^8^. Works aimed at defining or helping to define reference intervals for MPO levels in health and disease conditions are very important, especially in patients with neurological disorders.

In the neonatal period, GSH is the most important endogenous non-enzymatic antioxidant. Several hypoxia and reoxygenation studies in mice have measured high levels of the GSH/oxidized glutathione (GSSG) ratio. GSH is one of the most abundant antioxidants in the body and important in the highly oxidative brain, as it protects the cell against reactive oxygen compounds, and a depletion of GSH will result in mitochondrial dysfunction^2^,^16^. Our results show a reduction in GSH from the hypoxia group in relation to to the control group, although there was no statistically significant difference (0.3481±0.0730 HG *versus* 0.4113±0.0890 CG).

It was analyzed in Down syndrome patients before and after antioxidant supplementation. Parisotto *et al.* 17 found reduction in SOD and CAT levels even after six months of intervention therapy, not significantly changing their values^17^. In our results, although there is no significant difference between the hypoxia and control groups, we saw a higher expression of SOD and CAT in the hypoxia group, albeit mild, when compared to the control group of animals.

Thoresen *et al*.^4^ evaluated moderate hypothermia as a therapeutic strategy for a model of hypoxic ischemic encephalopathy in piglets and found no significant difference between the evaluated groups.

Huun *et al*.^16^ also did not find significant differences in the basal physiology of the groups before the hypoxic insult and portrayed the good pH recovery of the piglets hours after the damage, demonstrating their resistance and good recovery, while producing the desired damages of the animal model hypoxic disease. These blood gas parameters recovered and were not statistically different from sham-operated piglets 4 h after reoxygenation^11^.

Liu *et al*.^5^ analyzed the oxidative stress of newborn piglets and after 48 h of recovery from hypoxia and reoxygenation. There was no difference in the GSH concentration in the piglet brain between the experimental groups. Although the group treated with N-acetylcysteine was greater than the control, the difference did not reach statistical significance^5^.

The mouse models whose mechanism and effect of hypoxia are studied are very diverse. A lot of variability was found, as well as different parameters such as the age of the mouse, the percentages of oxygen to which they are submitted to and the time they are exposed to different concentrations of oxygen. There are models that use an oxygen deprivation chamber, without a previous ischemic procedure, with a variation in oxygen concentration between 4 and 14% O_2_. The exposure time is the object of study, also ranging from 4 min to 48 hours, describing mild lesions and avoiding non-physiological occlusion of the common carotid artery^2^.

The most frequently used large animal models of perinatal asphyxia include partial or complete asphyxia at parturition, umbilical cord occlusion, hypoperfusion, and hypoxia in newborn piglets. The need for continuous research to establish the ideal model and find adjuvant neuroprotection therapy is highlighted^18^.

## Conclusions

The present study contributes to the use of the piglet as an animal model of neonatal hypoxia and brain damage, although it did not show any significant difference compared to the analyzed control group, in the brain of newborn piglets. More studies are needed to improve the harm induction protocol, as well as the development of studies of different therapeutic options, as observed in several countries. As for the characterization of oxidative stress, early diagnosis increases the chances of treatment success, but continuous research on new biomarkers, as well as their status, is necessary, whether under normal or pathological conditions developed in experimental disease models.

## References

[1] Paiva LA, Silva IS, Souza AS, Cassino PC. (2017). Pulmonary oxidative stress in diabetic rats exposed to
hyperoxia. Acta Bras Cir.

[2] Torres-Cuevas I, Corral-Debrinski M, Gressens P. (2019). Brain oxidative damage in murine models of neonatal
hypoxia/ischemia and reoxygenation. Free Radic Biol Med.

[3] Li P, Stetler RA, Leak RK, Shi Y, Li Y, Yu W, Bennett MVL, Chen J. (2018). Oxidative stress and DNA damage after cerebral ischemia:
potential therapeutic targets to repair the genome and improve stroke
recovery. Neuropharmacology.

[4] Thoresen M, Satas S, Løberg EM, Whitelaw A, Acolet D, Lindgren C, Penrice J, Robertson N, Haug E, Steen PA. (2001). Twenty-four hours of mild hypothermia in unsedated newborn pigs
starting after a severe global hypoxic-ischemic insult is not
neuroprotective. Pediatr Res.

[5] Liu JQ, Lee TF, Chen C, Bagim DL, Cheung PY. (2010). N-acetylcysteine improves hemodynamics and reduces oxidative
stress in the brains of newborn piglets with hypoxia-reoxygenation
injury. J Neurotrauma.

[6] Zhao M, Zhu P, Fujino M, Zhuang J, Guo H, Sheikh I, Zhao L, Li XK. (2016). Oxidative stress in hypoxic-ischemic encephalopathy: molecular
mechanisms and therapeutic strategies. Int J Mol Sci.

[7] Campos MTG, Leme FOP (2018). Estresse oxidativo: fisiopatogenia e diagnóstico
laboratorial. Pubvet.

[8] Vellosa JCR, Parabocz GC, Manente FA, Ribas JT, Lima LW (2013). Alterações metabólicas e inflamatórias em condições de estresse
oxidativo. Rev Ciênc Farm Básica Apl.

[9] Eiby YA, Wright LL, Kalanjati VP, Miller SM, Bjorkman ST, Keates HL, Lumbers ER, Colditz PB, Lingwood BE (2013). A pig model of the preterm neonate: anthropometric and
physiological characteristics. PLoS One.

[10] Schulz S, Reulecke S, Eiselt M, Schwab K, Witte H, Walter B, Bauer R, Voss A. (2011). Quantification of compensatory processes of postnatal hypoxia in
newborn piglets applying short-term nonlinear dynamics
analysis. Biomed Eng Online.

[11] Nemeth J, Toth-Szuki V, Varga V, Kovacs V, Remzso G, Domoki F. (2016). Molecular hydrogen affords neuroprotection in a translational
piglet model of hypoxic-ischemic encephalopathy. J Physiol Pharmacol.

[12] Rao TS, Currie JL, Shaffer AF, Isakson PC. (1993). Comparative evaluation of arachidonic acid (AA)- and
tetradecanoylphorbol acetate (TPA)-induced dermal
inflammation. Inflammation.

[13] Kyng KJ, Skajaa T, Kerrn-Jespersen S, Andreassen CS, Bennedsgaard K, Henriksen TB. (2015). A piglet model of neonatal hypoxic-ischemic
encephalopathy. J Vis Exp.

[14] Maia FES, Paiva MBM, Clemente CJE (2016). Suporte ventilatório e o estresse oxidativo em
prematuro. Rev Aten Saúde.

[15] Parisotto EB, Vidal V, García-Cerro S, Lantigua S, Wilhelm Filho, Sanchez-Barceló EJ, Martínez-Cué C, Rueda N (2016). Chronic melatonin administration reduced oxidative damage and
cellular senescence in the hippocampus of a mouse model of Down
syndrome. Neurochem Res.

[16] Huun MU, Garberg H, Løberg EM, Escobar J, Martinez-Orgado J, Saugstad OD, Solberg R. (2018). DHA and therapeutic hypothermia in a short-term follow-up piglet
model of hypoxia-ischemia: effects on H+MRS biomarkers. PLoS One.

[17] Parisotto EB, Giaretta AG, Zamoner A, Moreira EA, Fröde TS, Pedrosa RC, Filho DW (2015). Persistence of the benefit of an antioxidant therapy in children
and teenagers with Down syndrome. Res Dev Disabil.

[18] Koehler RC, Yang ZJ, Lee JK, Martin LJ. (2018). Perinatal hypoxic-ischemic brain injury in large animal models:
Relevance to human neonatal encephalopathy. J Cereb Blood Flow Metab.

